# Development of New Carbon Resources: Production of Important Chemicals from Algal Residue

**DOI:** 10.1038/s41598-017-00979-y

**Published:** 2017-04-12

**Authors:** Sho Yamaguchi, Yuuki Kawada, Hidetaka Yuge, Kan Tanaka, Sousuke Imamura

**Affiliations:** 1 Department of Chemical Science and Engineering, School of Materials and Chemical Technology, Tokyo Institute of Technology, 4259-G1-14 Nagatsuta-cho, Midori-ku, Yokohama Kanagawa, 226-8502 Japan; 2 Department of Chemistry, School of Science, Kitasato University, 1-15-1 Kitasato, Minami-ku, Sagamihara Kanagawa, 252-0373 Japan; 3 Laboratory for Chemistry and Life Science, Institute of Innovative Research, Tokyo Institute of Technology, 4259-R1-30 Nagatsuta-cho, Midori-ku, Yokohama Kanagawa, 226-8502 Japan; 4 Core Research for Evolutional Science and Technology (CREST), Japan Science and Technology Agency (JST), Saitama, 332-0012 Japan

## Abstract

Algal biomass has received attention as an alternative carbon resource owing not only to its high oil production efficiency but also, unlike corn starch, to its lack of demand in foods. However, algal residue is commonly discarded after the abstraction of oil. The utilization of the residue to produce chemicals will therefore increase the value of using algal biomass instead of fossil fuels. Here, we report the use of algal residue as a new carbon resource to produce important chemicals. The application of different homogeneous catalysts leads to the selective production of methyl levulinate or methyl lactate. These results demonstrate the successful development of new carbon resources as a solution for the depletion of fossil fuels.

## Introduction

Many important chemicals are made from fossil fuels. For example, petroleum oil is widely used as gasoline, heating oil and a raw material for plastics. Furthermore, technology has recently been developed to obtain various chemicals from hydrocarbons in natural gas, such as methane and ethane. However, owing to the depletion of fossil fuels, the development of new carbon resources is a considerable global issue to be overcome. Accordingly, carbohydrates derived not only from woody biomass (i.e., cell wall polysaccharides such as cellulose) but also from crop biomass (i.e., storage polysaccharides such as glycogen and starch) have been considered as alternative carbon resources^1–8^. Methods for converting carbohydrates into several important chemicals have been developed, which are expected to have a consequent effect on reducing carbon dioxide emissions. However, as the global population continues to increase, the use of farmland to grow the crop biomass required for the production of carbon resources is clearly unrealistic, as the limited areas of farmland on Earth will be devoted to the production of food. Furthermore, large-scale deforestation to obtain sufficient woody biomass would lead to environmental destruction. Thus, the establishment of a more efficient carbon resource supply system that does not require farmland or cause environmental destruction is required.

As an alternative to woody and crop biomass-based systems, microalgae have been focused on as a new biomass production system because the biomass productivity per unit time and per unit area is predominantly high^9^. Additionally, algae can be propagated industrially without using farmland^10, 11^. The main characteristic of algae is high productivity of oil and carbohydrates, which consist mainly of starch, in cells under unfavorable growth conditions, such as nutrient deficiency^9^. Recently, the importance of oil resources derived from algae has been accepted because the oil has been used as biojet fuel or biodiesel^12–17^. However, unlike cellulose, the utilization of starch remaining in algal cells after oil extraction has been overlooked, and, therefore, we have focused on the conversion of this starch into important chemicals.

Dusselier *et al*. reported the one-pot synthesis of alkyl lactate or alkyl levulinate, which are important raw materials, from cellulose^18^. For example, two molecules of lactic acid can be dehydrated to the lactone lactide and subsequently polymerized to either atactic or syndiotactic polylactide, which are biodegradable polyesters^19^. On the other hand, levulinic acid is used not only as a precursor for pharmaceuticals, plasticizers, and various other additives but also as a building block or starting material for a wide number of compounds^20–23^. In the reported method, the use of homogeneous Sn^II^ triflate led to an increase in the yield of alkyl lactate, whereas a balance between Lewis (Sn) and Brønsted acids (trifluoromethanesulfonic acid; TfOH) could be used to influence the selectivity of each product. Glucose is the building unit of both cellulose and starch, although the binding form (α or β) and position of the glycosidic linkages are different, and so the methods in this impressive report should be adaptable for the successful utilization of algal residue. Herein, we report the efficient conversion of starch remaining in algal cells into methyl lactate or methyl levulinate. Moreover, we examined the differences in the reactivity of authentic starch and algal residue. Thus, we propose algal residue as a new alternative carbon resource to fossil fuels.

## Results

### Screening of catalysts for the conversion of authentic starch to important chemicals

**Figure 1:**
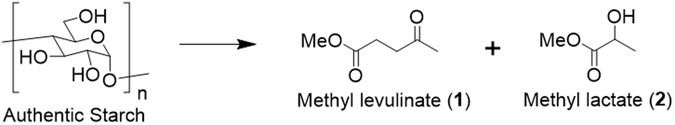
Conversion of an authentic starch sample into methyl levulinate (**1**) and methyl lactate (**2**).

In this work, the conversion of authentic starch to important chemicals was first accomplished in methanol at 160 °C for 24 h (Fig. 1). In an effort to increase the yield of the desired products methyl levulinate (**1**) and methyl lactate (**2**), a range of experiments employing different catalysts was conducted. The catalysts used, and the yields of the identified products, **1** and **2**, are given in Table 1.

**Table 1:** Conversion of an authentic starch sample into methyl levulinate (**1**) and methyl lactate (**2**) using various catalysts^a^.

Entry	Catalyst	**1** (%)^c^	**2** (%)^c^
1	Sn(OTf)_2_	48	2
2	Sn(CH_3_COO)_2_	0	0
3^b^	SnO	0	0
4^b^	SnO_2_	0	1
5	SnCl_2_∙2H_2_O	0	4
6	SnCl_4_∙5H_2_O	0	4
7^b^	SnCl_4_∙5H_2_O	3	0
8	SnBr_4_	5	8
9^b^	SnBr_4_	7	27
10	SnI_4_	3	4
11^b^	SnI_4_	3	20
12	No catalyst	0	0

^a^Reaction conditions: starch (50 mg), methanol (5.0 mL), catalyst (0.024 mmol), naphthalene (0.156 mmol), Ar (5 atm), 24 h, 160 °C. All reactions were performed twice, and the yields of **1** and **2** are average values. ^b^Catalyst (0.24 mmol). ^c^Yields (%) of each product are carbon-based. The amounts of **1** and **2** produced were determined by ^1^H NMR analysis.

In entry 1, when Sn(OTf)_2_ was applied as the catalyst, **1** and **2** were obtained in 48% and 2% yields, respectively. According to the report of Dusselier *et al*.^18^, application of Sn(OTf)_2_ to the degradation of cellulose leads to selective conversion to alkyl lactate. Therefore, this result shows that the reactivities of cellulose and starch are a quite different, even though both cellulose and starch are composed of glucose monomers. The subsequent use of Sn(OAc)_2_, SnO, and SnO_2_, which possess different counter anions and Sn oxidation states, did not afford either **1** or **2**, as the conversion of starch was very low (entries 2–4). Then, the use of tin chlorides was examined because tin chlorides have high activities for the retro-aldol reaction of carbohydrates^24^ followed by a 1,2-hydride shift and dehydration^25–28^. In entries 5 and 6, when divalent and tetravalent tin chlorides were applied, **1** was not obtained and **2** was obtained in low yields. As alkyl lactate is likely obtained from glucose by using divalent and tetravalent tin chlorides, we predicted that the conversion of starch to glucose (i.e., disconnection of a glycosidic linkage) did not proceed smoothly. Based on this result, we applied ten times the amount of SnCl_4_∙5H_2_O with a view of accelerating the degradation of starch to glucose monomers. However, the yields of **1** and **2** did not increase (entry 7). Although other potential useful products were not obtained, there was little starch left in the reaction mixture. Subsequently, changing the counter halogen atom was examined. Surprisingly, when SnBr_4_ was used, the product selectivity was inverted, and the use of ten times the amount of SnBr_4_ dramatically increased the yield of **2**, i.e. 8% and 27% yields, respectively (entries 8 and 9). The use of SnI_4_ afforded a similar result, with **2** obtained in 20% yield (entries 10 and 11). These results show that the catalyst had a clear influence on product selectivity. That is, the use of Sn(OTf)_2_ and SnBr_4_ leads to the selective production of **1** and **2**, respectively. Note that neither product was obtained without a catalyst (entry 12).

The data for optimizing the reaction conditions, including reaction temperature and reaction time, are given in the supporting information. At a reaction temperature of 160 °C, the yield of **1** was maximized, whereas the yield of **2** increased with reaction temperature (Figure S1). Moreover, constant yields of **1** and **2** were obtained at a reaction time of 24 h using Sn(OTf)_2_ or SnBr_4_ (Figure S2).

As shown in Table 1, entry 1, the use of Sn(OTf)_2_ leads to selective conversion of starch to **1**. We next examined the use of other metal triflate catalysts (Table 2). When Cu(OTf)_2_, AgOTf, Sc(OTf)_3_, and In(OTf)_3_ were used^29^, no **2** was obtained, allowing **1** to be obtained with high selectively (entries 2–5). Furthermore, when TfOH was applied alone as a Brønsted acid, **1** was afforded in the highest yield (entry 6). These results indicate that the production of **1** in this reaction system does not require a metal center as a Lewis acid, but does need TfOH as a Brønsted acid.

**Table 2:** Conversion of an authentic starch sample into methyl levulinate (**1**) and methyl lactate (**2**) using metal triflate catalysts^a^.

Entry	Catalyst	1 (%)^c^	2 (%)^c^
1	Sn(OTf)_2_	48	2
2	Cu(OTf)_2_	47	1
3	AgOTf	36	<1
4	Sc(OTf)_3_	39	1
5	In(OTf)_3_	33	1
6^b^	TfOH	53	<1

^a^Reaction conditions: starch (50 mg), methanol (5.0 mL), catalyst (0.024 mmol), naphthalene (0.156 mmol), Ar (5 atm), 24 h, 160 °C. All reactions were performed twice, and the yields of **1** and **2** are average values. ^b^Catalyst (0.048 mmol). ^c^Yields (%) of each product are carbon-based. The amounts of **1** and **2** produced were determined by ^1^H NMR analysis.

**Figure 2:**
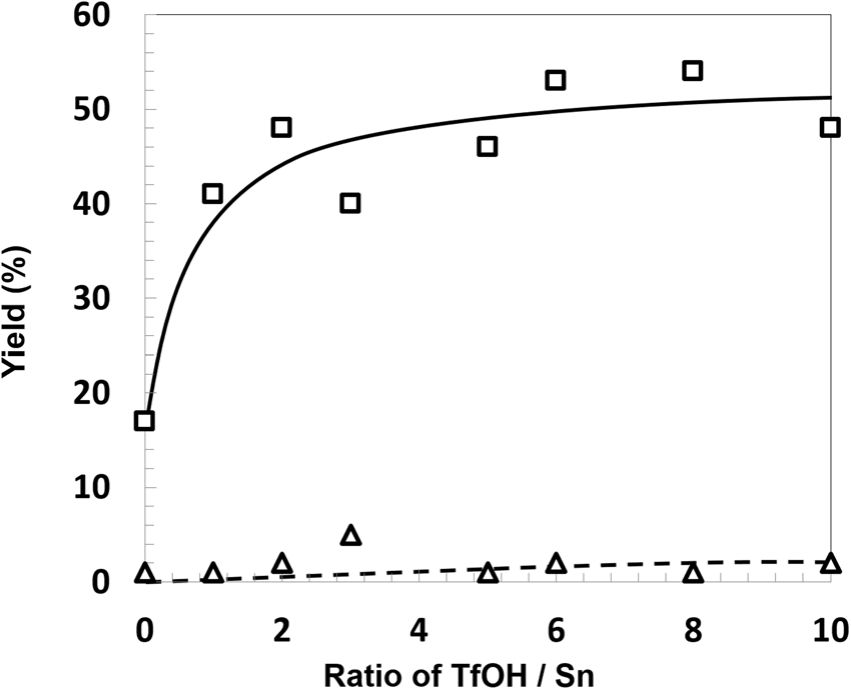
Influence of the ratio of Sn to OTf ions on the yields of **1** and **2**. Reaction conditions: starch (50 mg), methanol (5.0 mL), Sn(OTf)_2_ (0.024 mmol), naphthalene (0.156 mmol), Ar (5 atm), 24 h, 160 °C.

We further investigated the influence of the ratio of Sn to OTf ions on the reaction activity. Quenching the triflate groups of Sn(OTf)_2_ or the addition of excess TfOH can change the ratio of Lewis and Brønsted acidity in the reaction mixture. As shown in Fig. 2, when the triflate groups were quenched, the yield of **1** decreased. In contrast, the addition of TfOH increased the yield of **1**, and above a Sn to OTf ion ratio of 1:6, the yield of **1** remained unchanged. These results confirmed that the use of Sn(OTf)_2_ did not afford **2** and indicated that the production of **1** was dependent on the amount of TfOH used as a Brønsted acid. When using cellulose^18^, the TfOH/Sn ratio has an influence on the yields of both **1** and **2**. Therefore, the experimental results in Fig. 2 reveal the difference in reactivity between cellulose and starch.

Finally, we examined the conversion of starch using metal catalysts with halogen atom counter anions. As shown in Table 1, entry 9, the use of SnBr_4_ provided **2** as the major product. Based on this result, other bromide metal catalysts were applied to the conversion of starch (Table 3). When ZnBr_2_ and CuBr_2_ were used, neither of the desired products (**1** and **2**) were obtained (entries 4 and 5), whereas the use of ten times the amount of CuBr_2_ afforded **1** and **2** in 13% and 5% yields, respectively (entry 6). Subsequently, the use of hydrogen chloride (HCl) or hydrogen bromide (HBr) without a metal center was examined (entries 7 and 8). When HCl was applied, the desired products were not obtained, whereas when HBr was applied, **1** and **2** were obtained in 21% and 10% yields, respectively. Overall, the results indicate that selective production of **1** requires only TfOH as a Brønsted acid, whereas the selective production of **2** needs both a tin metal center as a Lewis acid and hydrogen bromide as a Brønsted acid. Interestingly, tin catalysts show specific activity for the selective production of **2**.

**Table 3:** Conversion of an authentic starch sample into methyl levulinate (**1**) and methyl lactate (**2**) using metal catalysts with halogen atom counter anions^a^.

Entry	Catalyst	1 (%)^e^	2 (%)^e^
1^b^	SnCl_4_∙5H_2_O	3	0
2^b^	SnBr_4_	7	27
3^b^	SnBr_2_	8	7
4	ZnBr_2_	0	0
5	CuBr_2_	4	2
6^b^	CuBr_2_	13	5
7^c^	HCl	0	1
8^d^	HBr	21	10

^a^Reaction conditions: starch (50 mg), methanol (5.0 mL), catalyst (0.024 mmol), naphthalene (0.156 mmol), Ar (5 atm), 24 h, 160 °C. All reactions were performed twice, and the yields of **1** and **2** are average values. ^b^Catalyst (0.24 mmol). ^c^Catalyst (0.048 mmol). ^d^Catalyst (0.96 mmol) in 25% AcOH. ^e^Yields (%) of each product are carbon-based. The amounts of **1** and **2** produced were determined by ^1^H NMR analysis.

### Conversion of algae into alkyl levulinate and alkyl lactate

**Figure 3:**
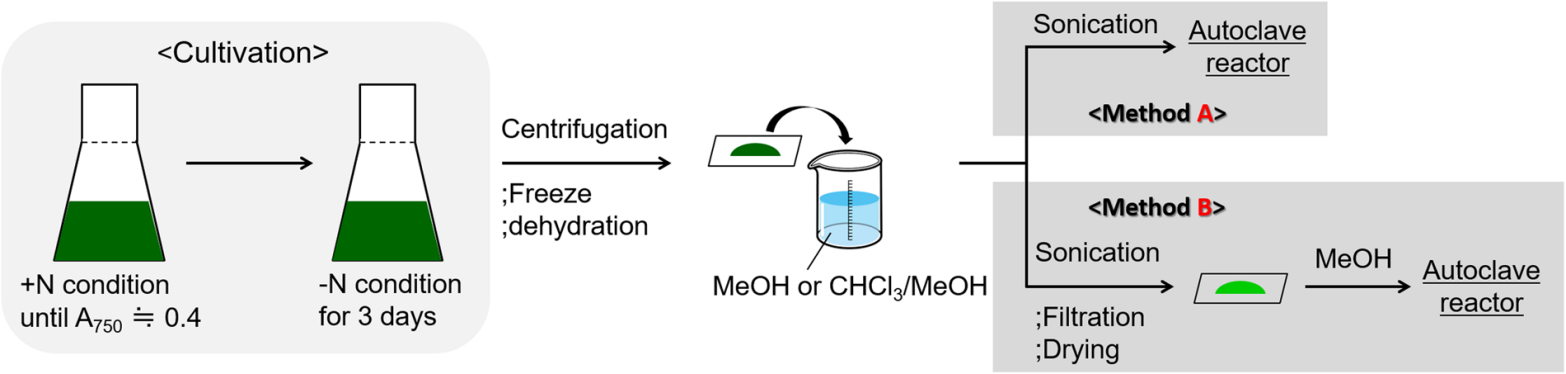
Method for the preparation of algal residue.

**Figure 4:**
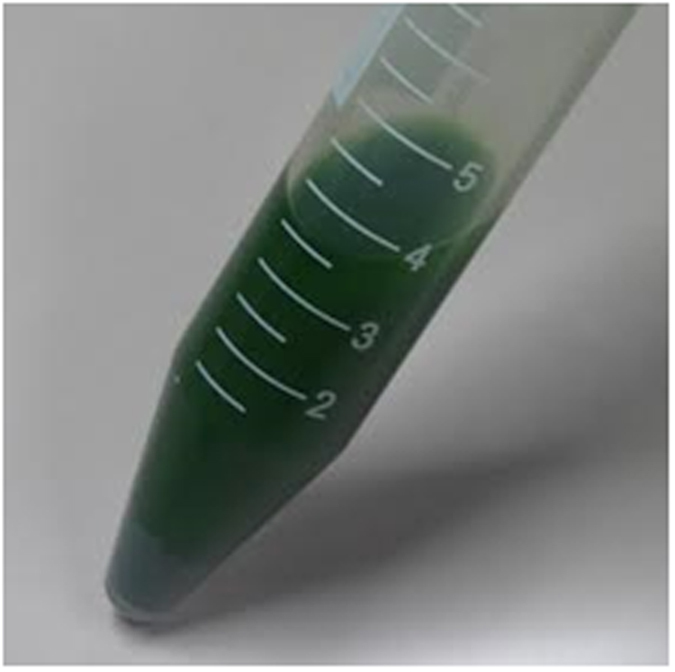
An image of the prepared sample.

As demonstrated above, authentic starch can be selectively converted into **1** or **2** by choosing the appropriate conditions. Based on these results, the reaction using algae was investigated. The cultivation conditions, reaction pretreatment, and quantification of neutral sugars are described in the Methods. Furthermore, the methods for the preparation of algae as a reaction substrate are illustrated in Fig. 3. After the cultivation of algae, centrifugation, freezing, and dehydration steps afforded a green powder containing mineral ions and a pigment extracted with methanol from the cells. Then, the powder was suspended in methanol, and sonication of this mixture provided the reaction substrate in methanol solution (Fig. 3, method A). When the sonication is carried out in methanol, the prepared substrate contains both oil and carbohydrates (Fig. 4). In this study, the yields of the desired products (**1** and **2**) were based on the amount of carbohydrates in algae, which was calculated according to the quantification of neutral sugars, as described in the Methods.

First, the reaction of the sample prepared based on Fig. 3, method A was investigated. Sn(OTf)_2_ was applied as the catalyst, in the same catalytic amount as when using authentic starch. As shown in Table 4, entry 1, the desired products **1** and **2** were not obtained. Based on this result, we hypothesized that the mineral ions and pigment contained in algae influenced the catalytic activity, likely through catalyst poisoning. Therefore, the sample obtained after sonication was filtered, washed with methanol, and then dried *in vacuo* (Fig. 3, method B) to remove the mineral ions and pigment. This sample was then used to examine the degradation of the algal carbohydrates. Using Sn(OTf)_2_, the desired products **1** and **2** were obtained in 37% and 9% yields, respectively (entry 2). The use of SnBr_4_ was also demonstrated, and **2** was predictably obtained as the major product; that is, **1** and **2** were obtained in 6% and 37% yields, respectively (entry 3, cf. Table 1, entry 9). Based on these results, optimizing the pretreatment to remove the mineral ions and pigment led to the production of the desired products.

**Table 4:** Conversion of algae into methyl levulinate (**1**) and methyl lactate (**2**) using Sn(OTf)_2_ or SnBr_4_^a^.

Entry	Methods	Solvent for suspension	Catalyst	**1** (%)^g^	**2** (%)^g^
1^b^	A	MeOH	Sn(OTf)_2_	0	0
2^c^	B	MeOH	Sn(OTf)_2_	37	9
3^d^	B	MeOH	SnBr_4_	6	37
4^e^	B	CHCl_3_/MeOH	Sn(OTf)_2_	30	7
5 ^f^	B	CHCl_3_/MeOH	SnBr_4_	4	31

^a^Reaction conditions: methanol (5.0 mL), catalyst (0.24 mmol), naphthalene (0.156 mmol), Ar (5 atm), 24 h, 160 °C. ^b^Catalyst (0.024 mmol). Algae (100 mg) containing 55.5 mg of carbohydrates. ^c^Algae (53.4 mg) containing 29.6 mg of carbohydrates. ^d^Algae (75.2 mg) containing 38.1 mg of carbohydrates. ^e^Algae (78.2 mg) containing 39.6 mg of carbohydrates. ^f^Algae (77.4 mg) containing 39.2 mg of carbohydrates. ^g^Yields (%) of each product are carbon-based. The amounts of **1** and **2** produced are based on the amount of carbohydrates and were determined by ^1^H NMR analysis. ^1^H NMR spectra of the reaction mixtures (entries 2 and 3) are shown in Figures S4 and S5, respectively.

However, the purpose of this study was to obtain the desired chemical products using algal residue after the abstraction of oil components, but peaks derived from the oil components of algae were observed in a detailed GC-MS analysis (Figure S3). Therefore, the sonication step was carried out in a mixed solvent (CHCl_3_/MeOH) to separate the oil components from the algal residue. When this residue was used for the conversion reaction, the results were similar to those obtained in the presence of the oil components; that is, when Sn(OTf)_2_ was applied, **1** and **2** were obtained in 30% and 7% yields, respectively (entry 4), whereas when SnBr_4_ was applied, **1** and **2** were obtained in 4% and 31% yields, respectively (entry 5). Based on these results, we predict that the total lipids in the cells did not have a detrimental effect on this reaction.

## Discussion

**Figure 5:**
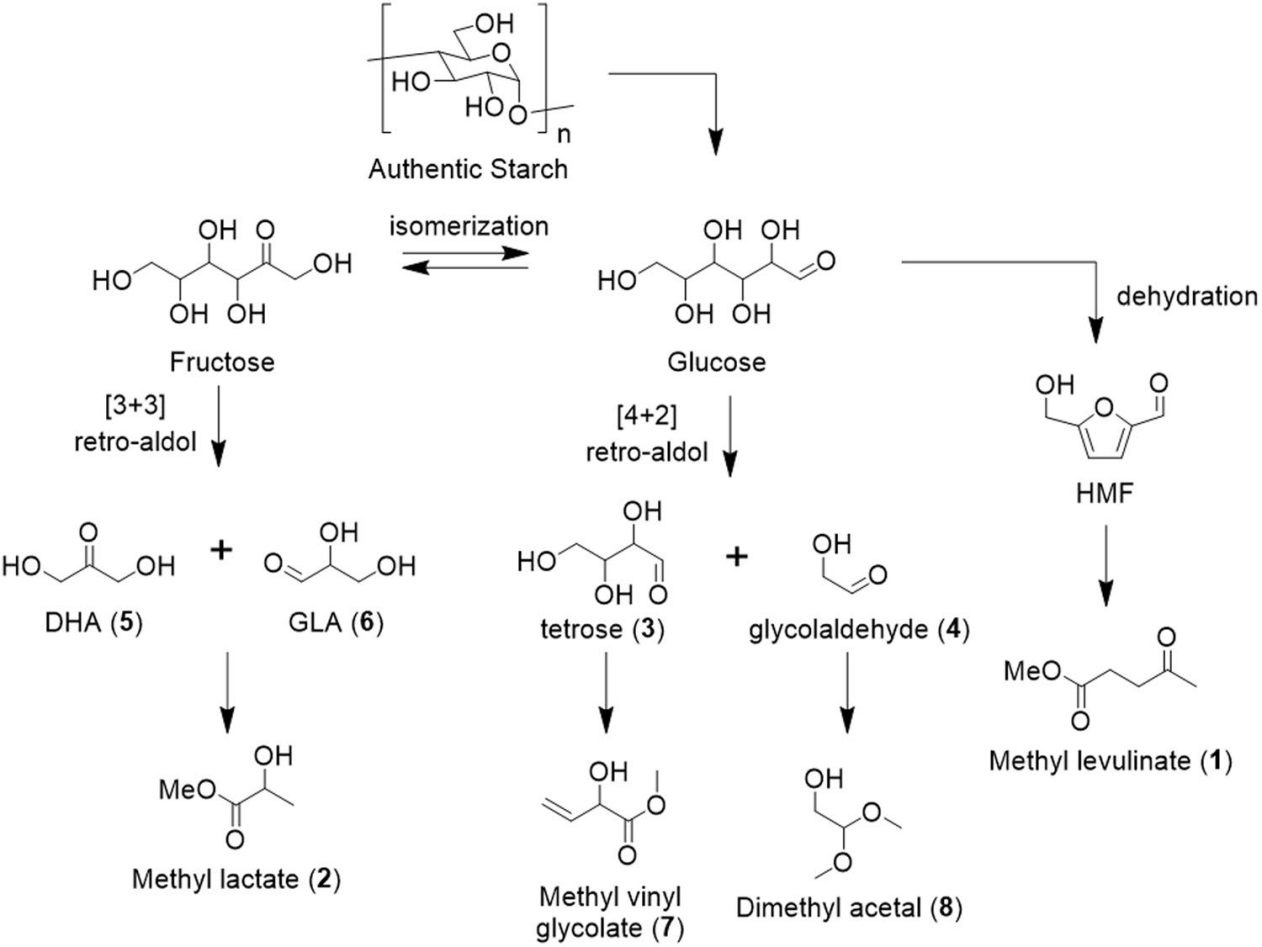
Transformation of starch into **1**, **2**, and several other important chemicals.

In this reaction system, alkyl levulinate and alkyl lactate were obtained by the degradation of not only an authentic starch sample, but also algae. The mechanism for formation of these products is explained based on Fig. 5. A glucose monomer, which is generated through the disconnection of a glycosidic bond in starch, is converted into tetrose (**3**) and glycolaldehyde (**4**) via a [4 + 2] retro-aldol reaction, whereas 1,3-dihydroxyacetone (**5**) and glyceraldehyde (**6**) are obtained via a [3 + 3] retro-aldol reaction. Based on a previous report, the [3 + 3] retro-aldol reaction seems to be preferential thermodynamically compared with the [4 + 2] retro-aldol reaction. In fact, methyl vinyl glycolate (**7**) and dimethyl acetal (**8**) generated from **3** and **4**, respectively, were not obtained^30, 31^. In contrast, the conversion of **5** or **6** into **2** proceeded to give alkyl lactate. Note that this pathway has been reported by Sasaki *et al*.^28^. On the other hand, when we applied Sn(OTf)_2_ as a catalyst, alkyl levulinate was obtained via the dehydration of glucose^29, 32–35^. This result is consistent with previous reports, i.e., a Brønsted acid effectively catalyzes a sequential dehydration reactions of glucose. Furthermore, the use of SnBr_4_ provided **2** selectively. However, this result is inconsistent with the conversion of 3- or 6-carbon sugars into **2**. SnCl_4_ has the best performance among tin halides (SnCl_4_, SnBr_4_, and SnI_4_) for the production of **2** from 3- or 6-carbon sugars^24–27^. As chloride has the largest electronegativity among halogen atoms, the strong Lewis acidity of Sn in SnCl_4_ seems to accelerate the retro aldol reaction, dehydration, and 1,2-hydride shift. On the other hand, when the conversion of starch or algae to **2** was examined, the use of SnCl_4_, SnBr_4_, and SnI_4_ afforded **2** in 0%, 27%, and 20% yields, respectively (Table 1, entries 7, 9, and 11). HCl as a Brønsted acid generated from SnCl_4_ has the lowest acidity among the three hydrogen halides, and therefore, we predicted that the glycosidic linkages were not cleaved smoothly. In the case of SnI_4_, although the strong Brønsted acid HI accelerates the cleavage of the glycosidic linkages, the Lewis acidity of Sn in SnI_4_ is lower than that in SnBr_4_ owing to differences in electronegativity. Consequently, the yield using SnBr_4_ is higher than that using SnI_4_.

To demonstrate the novelty and quality of our results for **1** and **2** production, the results from previous reports on the conversion of several sugars into **1** and **2** are summarized in Table 5. In entry 2, when Sn(OTf)_2_ was applied to the degradation of cellulose, similar amounts of **1** and **2** were obtained^18^. This result indicates that a balance between Sn as a Lewis acid and TfOH as a Brønsted acid is key to the degradation of cellulose, although the conversion of algae with Sn(OTf)_2_ afforded **1** selectively. As shown in entries 3 and 4, when homogeneous and heterogeneous catalysts were applied to the degradation of cellulose, the homogeneous catalyst led to higher productivity^36, 37^. On the other hand, there are few examples of the conversion of polysaccharides into **2**, and almost all reports on the synthesis of **2** focus on the use of mono- and disaccharides (entries 5–8)^24, 28, 38, 39^. Thus, we believe that the catalytic performance of SnBr_4_ for the conversion of polysaccharides into **2** is excellent.

**Table 5:** Previous studies on the conversion of carbohydrates into methyl levulinate (**1**) and methyl lactate (**2**).

Entry	Substrate	Temperature (°C)	Time (h)	Catalyst	Yield (%)		ref
					**1**	**2**	
1	Algae (this study)	160	24	Sn(OTf)_2_	37^a^	9^a^	—
				SnBr_4_	6^a^	37^a^	—
2	Microcrystalline avicel cellulose	200	2	Sn(OTf)_2_	23^a^	24^a^	18
3	Cellulose	190	5	H_2_SO_4_	55	—	36
4	Cellulose	200	20	H-USY	13	—	37
5	Glucose	160	24	Sn-Beta	—	43^a^	24
6	1,3-Dihydroxyacetone	90	3	SnCl_4_∙5H_2_O	—	82	28
7	1,3-Dihydroxyacetone	115	24	H-USY	—	96	38
8	Sucrose	170	16	Alkali salts + tin-containing silicate	—	ca. 75	39

^a^Yields are carbon-based.

## Conclusion

In conclusion, we achieved the selective conversion of algae to methyl levulinate and methyl lactate, which are important for the production of fine chemicals. Using an authentic starch sample, various homogeneous catalysts were examined to optimize the selective production of methyl levulinate or methyl lactate. Subsequently, these optimized conditions were applied for the successful conversion of algae. The results of this study show that algae can be utilized not only for oil production, but also as a carbon resource for important chemicals. Therefore, we suggest the utilization of algae biomass as a new alternative carbon resource to fossil fuels.

## Methods

### General

All materials were obtained from Sigma Aldrich, TCI, Wako Chemicals, or Kanto Kagaku and used as received. In a typical reaction (Table 1 entry 1), a 50 mL stainless steel autoclave with mechanical stirring and controlled heating was loaded with 50 mg of starch, 0.024 mmol of Sn(OTf)_2_, 0.020 g of naphthalene (internal standard), and 5 mL of methanol. The reactor was sealed, flushed 3 times with 5 atm of Ar, and finally pressurized with 5 atm of Ar. After stirring at 160 °C for 24 h under Ar, the reactor was cooled under ambient air. When the reactor reached room temperature, the pressure returned to 5 atm, indicating little or no formation of gaseous products.

To quench intrinsic triflate groups in the Sn(OTf)_2_ complex, the catalyst was dissolved in methanol and a precise amount of Dowex Monosphere 550 A anion exchange resin (based on a quaternary amine) in its OH^−^ form was added. After stirring for 30 min at room temperature, the solution was separated from the beads and transferred to the reactor. To add extrinsic trifluoromethanesulfonic acid (TfOH), a solution of TfOH in methanol was prepared and added to the reaction mixture as the solvent. The quenching capacity of the resin (after complete exchange under excess NaOH and thorough washing) was determined by titration, and the anion exchange capacity was approximated as 1.5 mmol g^−1^.

The reaction products were identified by ^1^H nuclear magnetic resonance (NMR) and gas chromatography-mass spectrometry (GC-MS). The NMR spectra were recorded on Bruker Biospin AVANCE 400 (400 MHz for ^1^H) or Bruker Biospin AVANCE 500 (500 MHz for ^1^H) instruments. The chemical shifts (ppm) are reported in parts per million (ppm), using tetramethylsilane as internal (0 ppm) reference, in CDCl_3_ solutions. The ^1^H NMR spectra were referenced to CDCl_3_ (7.26 ppm) as an external standard. Multiplicities are reported using the following abbreviations: *s*: singlet, *d*: doublet, *t*: triplet, *q*: quartet, *m*: multiplet, *br*: broad, *J*: coupling constant in Hz. A Shimadzu QP2010 Plus instrument equipped with a TC-1 column was used for the GC-MS analysis, using the following temperature program: i) 2 min at 323 K, ii) linear ramp of 10 K min^−1^ to 553 K, and iii) 20 min at 553 K.

Product yields were calculated based on the internal standard naphthalene, and were calculated based on the total input of carbon of the carbohydrate feed, not taking into account solvent-derived methyl groups in the products. Considering the co-formation of 1 (methyl) formate for every levulinate, the maximum obtainable (theoretic) yield in our calculations for methyl levulinate (**1**) (5/6 carbons) corresponds to 83.3%. In Table 4, yields of each product are calculated on the basis that the amount of carbohydrates, including all-carbon monosaccharides, in algae are quantitated.

### The cultivation conditions

Algal strain and growth conditions was shown here. Briefly, *Cyanidioschyzon merolae* 10D wild-type^40^ was grown at 40 °C under continuous white light (50 μmol m^−2^ s^−1^) in liquid MA2 medium^41^, bubbled by air supplemented with 2% CO_2_. For nitrogen-depleted conditions to accumulate starch, *C*. *merolae* cells were grown until 0.43 absorbance units at A_750_ and were collected by centrifugation (5,000 g, room temperature, 15 minutes), and gently resuspended in the same volume of nitrogen free medium^42^. After 3 days incubation, the cells were harvested by centrifugation (1,600 g, room temperature, 5 minutes), frozen immediately in liquid nitrogen, and freeze-drying until use.

### Quantification of neutral sugars

The method is well suited to identify neutral sugars in polysaccharides, including glycogen and starch, but detects virtually all classes of carbohydrates^43^. Cell culture pellets of 0.25–1 mL were treated with 10% SDS (Wako) and washed with 80% ethanol (Wako) warmed to 50 °C. The resulting starch pellet was solubilized by boiling for 15 min in 75 μL of distilled water. After cooling to room temperature, 125 μL of 60% perchloric acid (Wako) was added and the mixture was vortexed for 15 min. Then, 300 μL of distilled water was added and the mixture was centrifuged at 20,000 × g for 15 min. Then, 200 μL of the sample was mixed with 200 μL of 5% aqueous phenol solution (Wako) in a microtube. Subsequently, 1 mL of concentrated sulfuric acid (Wako) was added rapidly to the mixture, which was incubated for 10 min at room temperature and then at 40 °C for 20 min for color development. The absorption value at 490 nm was then recorded using a spectrophotometer (Bio-Rad Model 680 Microplate). Glucose (Wako) was used to produce a standard curve.

### Reaction pretreatment

For the preparation of samples in Table 4, about 53.4–100 mg of freeze-drying the cells were suspended in 4 ml of methanol, and uniformly dispersed by a sonication (BRANSON SONIFIER 250, output level of 1.0, Duty 50, 1 min, 12 times). In case of preparation of de-oiled alga, about 53.4–100 mg of the freeze-drying the cells were suspended in 600 μl of distilled water and 3 ml of chloroform:methanol (1:2), uniformly dispersed as mentioned above, and incubated at room temperature for 45 minutes. After incubation, the mixture was centrifuged (180 g, room temperature, 3 minutes) and discarded the supernatant. The collected pellet was resuspended in 4 ml of chloroform:methanol (1:2) to remove residual oil.

## Electronic supplementary material

supplementary info

## References

[1] Huber GW, Iborra S, Corma A (2006). Synthesis of transportation fuels from biomass: chemistry, catalysts, and engineering. Chem. Rev..

[2] Corma A, Iborra S, Velty A (2007). Chemical routes for the transformation of biomass into chemicals. Chem. Rev..

[3] Bozell JJ, Peterson GR (2010). Technology development for the production of biobased products from biorefinery carbohydrates–the US Department of Energy's “Top 10” revisited. Green Chem..

[4] Marshall AL, Alaimo PJ (2010). Useful products from complex starting materials: common chemicals from biomass feedstocks. Chem. Eur. J.

[5] Pileidis F, Titirici M (2016). Levulinic acid biorefineries: new challenges for efficient utilization of biomass. ChemSusChem.

[6] Yamaguchi S, Baba T (2016). A novel strategy for biomass upgrade: cascade approach to the synthesis of useful compounds via C-C bond formation using biomass-derived sugars as carbon nucleophiles. Molecules.

[7] Yamaguchi S, Baba T (2016). Cascade approach to the synthesis of useful compounds using various natural carbon resources. J. Synth. Org. Chem. Jpn.

[8] Binder JB, Raines RT (2009). Simple chemical transformation of lignocellulosic biomass into furans for fuels and chemicals. J. Am. Chem. Soc..

[9] Chisti Y (2007). Biodiesel from microalgae. Biotechnol. Adv..

[10] Rosenberg JN, Oyler GA, Wilkinson L, Betenbaugh MJ (2008). A green light for engineered algae: redirecting metabolism to fuel a biotechnology revolution. Curr. Opin. Biotechnol..

[11] Sheehan J (2009). Engineering direct conversion of CO_2_ to biofuel. Nat. Biotechnol..

[12] Spolaore P, Joannis-Cassan C, Duran E, Isambert A (2006). Commercial applications of microalgae. J. Biosci Bioeng..

[13] Ragauskas AJ (2006). The path forward for biofuels and biomaterials. Science.

[14] Wackett LP (2008). Biomass to fuels via microbial transformations. Curr. Opin. Chemical Biotechnol.

[15] Atsumi S, Higashide W, Liao JC (2009). Direct photosynthetic recycling of carbon dioxide to isobutyraldehyde. Nat. Biotechnol..

[16] Dexter J, Fu P (2009). Metabolic engineering of cyanobacteria for ethanol production. Energy Environ. Sci..

[17] Chen CY, Yeh KL, Aisyah R, Lee DJ, Chang JS (2011). Cultivation, photobioreactor design and harvesting of microalgae for biodiesel production: a critical review. Bioresour Technol.

[18] Dusselier M, Clercq R, Cornelis R, Sels BF (2017). Tin triflate-catalyzed conversion of cellulose to valuable (α-hydroxy-) esters. Catalysis Today.

[19] Dusselier M, Wouwe PV, Dewaele A, Makshina E, Sels BF (2013). Lactic acid as a platform chemical in the biobased economy: the role of chemocatalysis. Energy Environ. Sci..

[20] Bozell JJ (2000). Production of levulinic acid and use as a platform chemical for derived products. Resour., Conserv. Recycl..

[21] Manzer LE (2004). Catalytic synthesis of α-methylene-γ-valerolactone: a biomass-derived acrylic monomer. Appl. Catal., A.

[22] Guo Y, Li K, Clark JH (2007). The synthesis of diphenolic acid using the periodic mesoporous H_3_PW_12_O_40_-silica composite catalysed reaction of levulinic acid. Green Chem..

[23] Geilen FMA (2010). Selective and flexible transformation of biomass-derived platform chemicals by a multifunctional catalytic system. Angew. Chem., Int. Ed..

[24] Holm MS, Saravanamurugan S, Taarning E (2010). Conversion of sugars to lactic acid derivatives using heterogeneous zeotype catalysts. Science.

[25] Yamaguchi S, Motokura K, Sakamoto Y, Miyaji A, Baba T (2014). Tin-catalyzed conversion of biomass-derived triose sugar and formaldehyde to α-hydroxy-γ-butyrolactone. Chem. Commun..

[26] Yamaguchi S (2015). Mechanistic studies on the cascade conversion of 1,3-dihydroxyacetone and formaldehyde into α-hydroxy-γ-butyrolactone. ChemSusChem.

[27] Yamaguchi S, Matsuo T, Motokura K, Miyaji A, Baba T (2016). Cascade synthesis of five-membered lactones using biomass-derived sugars as carbon nucleophiles. Chem. Asian J..

[28] Hayashi Y, Sasaki Y (2005). Tin-catalyzed conversion of trioses to alkyl lactates in alcohol solution. Chem. Commun..

[29] Tominaga K, Mori A, Fukushima Y, Shimada S, Sato K (2011). Mixed-acid systems for the catalytic synthesis of methyl levulinate from cellulose. Green Chem..

[30] Dusselier M (2013). Mechanistic insight into the conversion of tetrose sugars to novel α-hydroxy acid platform molecules. ChemCatChem.

[31] Dusselier M (2013). Toward functional polyester building blocks from renewable flycolaldehyde with Sn cascade catalysis. ACS Catal.

[32] Thomas RW, Schuette HA (1931). Studies on levulinic acid. I. its preparation from carbohydrates by digestion with hydrochloric acid under pressure. J. Am. Chem. Soc..

[33] Seri K, Sakaki T, Shibata M, Inoue Y, Ishida H (2002). Lanthanum(III)-catalyzed degradation of cellulose at 250 °C. Bioresour. Technol.

[34] Takeuchi Y, Fangming J, Tohji K (2008). Acid catalytic hydrothermal conversion of carbohydrate biomass into useful substances. J. Mater. Sci..

[35] Peng LP (2010). Catalytic conversion of cellulose to levulinic acid by metal chlorides. Molecules.

[36] Wu X, Fu J, Lu X (2012). One-pot preparation of methyl levulinate from catalytic alcoholysis of cellulose in near-critical methanol. Carbohydr. Res..

[37] Saravanamurugan S, Riisager A (2013). Zeolite catalyzed transformation of carbohydrates to alkyl levulinates. ChemCatChem.

[38] West RM (2010). Zeolite H-USY for the production of lactic acid and methyl lactate from C3-sugars. Journal of Catalysis.

[39] Taarning E (2009). Zeolite-Catalyzed Isomerization of Triose Sugars. ChemSusChem.

[40] Kuroiwa T (1998). The primitive red algae *Cyanidium caldarium* and *Cyanidioschyzon merolae* as model system for investigating the dividing apparatus of mitochondria and plastids. Bioessays.

[41] Imamura S (2010). Nitrate assimilatory genes and their transcriptional regulation in a unicellular red alga *Cyanidioschyzon merolae*: genetic evidence for nitrite reduction by a sulfite reductase-like enzyme. Plant Cell Physiol..

[42] Imamura S (2009). R2R3-type MYB transcription factor, CmMYB1, is a central nitrogen assimilation regulator in *Cyanidioschyzon merolae*. Proc. Natl. Acad. Sci. USA.

[43] Dubois M, Gilles KA, Hamilton JK, Rebers PA, Smith F (1956). Colorimetric method for determination of sugars and related substances. Anal. Chem..

