# Supplementary material for: Development of New Carbon Resources: Production of Important Chemicals from Algal Residue
Source: Sci Rep. 2017 Apr 12;7:855. doi: 10.1038/s41598-017-00979-y (PMC5429806; doi:10.1038/s41598-017-00979-y)
Supplement: Supplementary file 1 — supplementary info [file 41598_2017_979_MOESM1_ESM.doc]

**Development of New Carbon Resources: Production of Important Chemicals from Algal Residue**

Sho Yamaguchi*^1^, Yuuki Kawada^2^, Hidetaka Yuge^2^, Kan Tanaka^3,4^, Sousuke Imamura^3,4^

^1^Department of Chemical Science and Engineering, School of Materials and Chemical Technology, Tokyo Institute of Technology, 4259-G1-14 Nagatsuta-cho, Midori-ku, Yokohama, Kanagawa 226-8502, Japan

^2^Department of Chemistry, School of Science, Kitasato University, 1-15-1 Kitasato, Minami-ku, Sagamihara, Kanagawa, 252-0373, Japan

^3^Laboratory for Chemistry and Life Science, Institute of Innovative Research, Tokyo Institute of Technology, 4259-R1-30 Nagatsuta-cho, Midori-ku, Yokohama, Kanagawa 226-8502, Japan

^4^Core Research for Evolutional Science and Technology (CREST), Japan Science and Technology Agency (JST), Saitama 332-0012, Japan

Corresponding Author

Dr. Sho Yamaguchi

E-mail:yamaguchi.s.ag@m.titech.ac.jp

TEL: +81-(0)-45-924-5417; FAX: +81-(0)-45-924-5441

**Table of Contents:**

Section Description Page

A Optimization of Reaction Temperature S3

B Optimization of Reaction Time S4

C GC-MS Analysis of Oil Components S6

D ^1^H NMR Charts of the Reaction Mixture (Table 4, Entries 2 and 3) S7

**A, Optimization of Reaction Temperature**


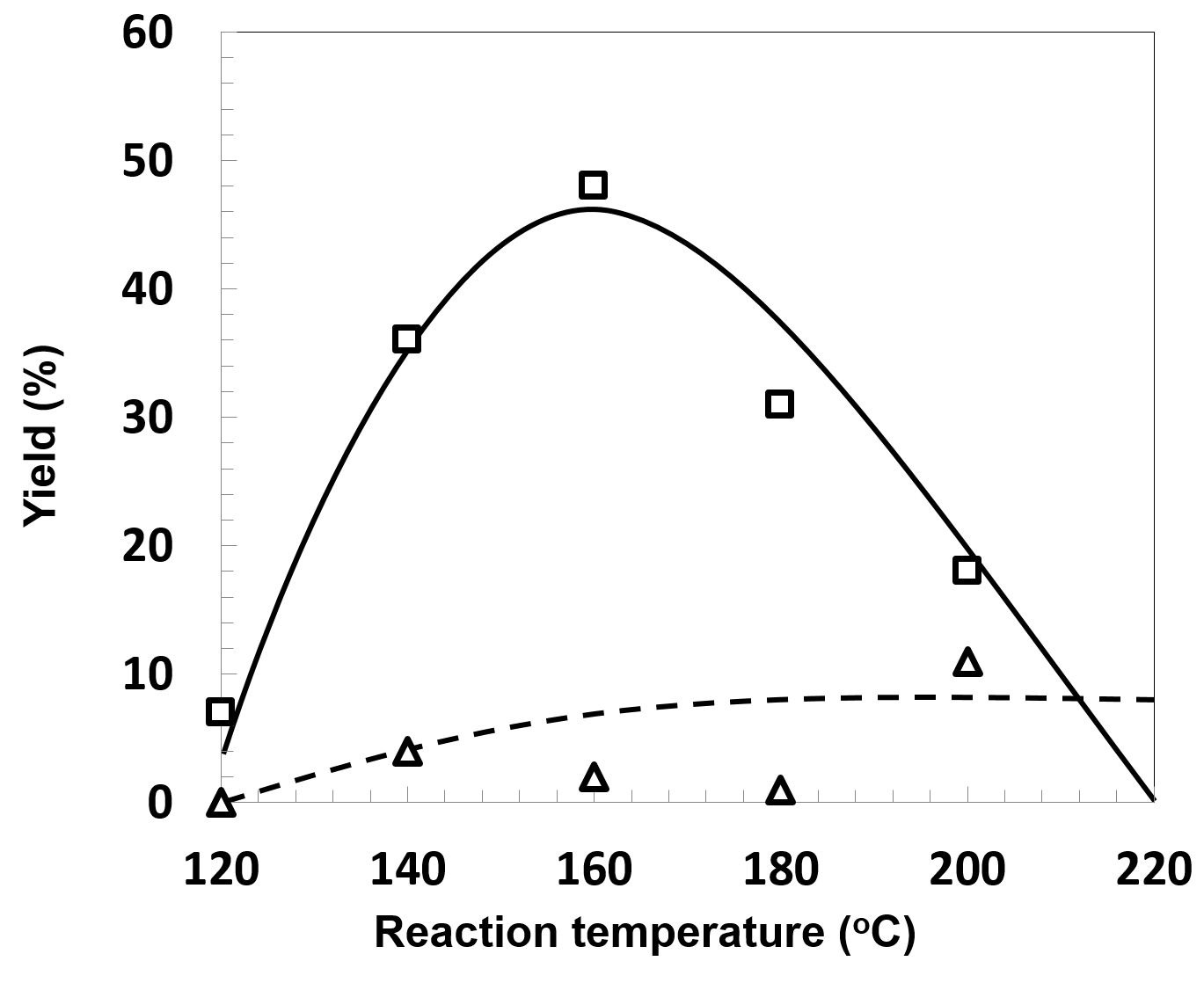


**Figure S1.** Effect of reaction temperature on the yields of methyl levulinate (**1**, □) and methyl lactate (**2**, △). Reaction conditions: starch (50 mg), methanol (5.0 mL), Sn(OTf)_2_ (0.024 mmol), naphthalene (0.156 mmol), Ar (5 atm), 24 h.

**B, Optimization of Reaction Time**


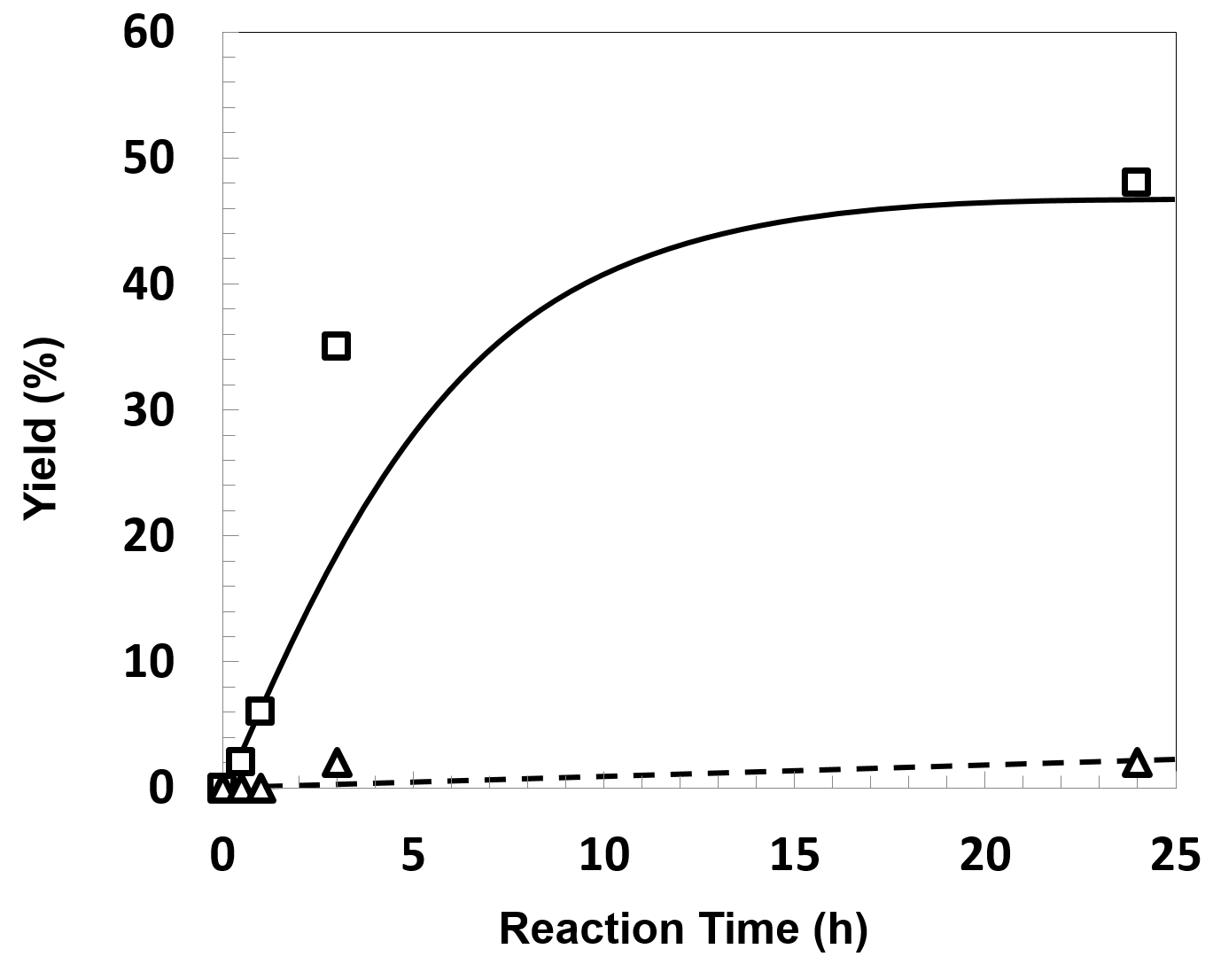


**Figure S2a.** Effect of reaction time on the yields of methyl levulinate (**1**, □) and methyl lactate (**2**, △) using Sn(OTf)_2_. Reaction conditions: starch (50 mg), methanol (5.0 mL), Sn(OTf)_2_ (0.024 mmol), naphthalene (0.156 mmol), Ar (5 atm), 24 h, 160 ^o^C.


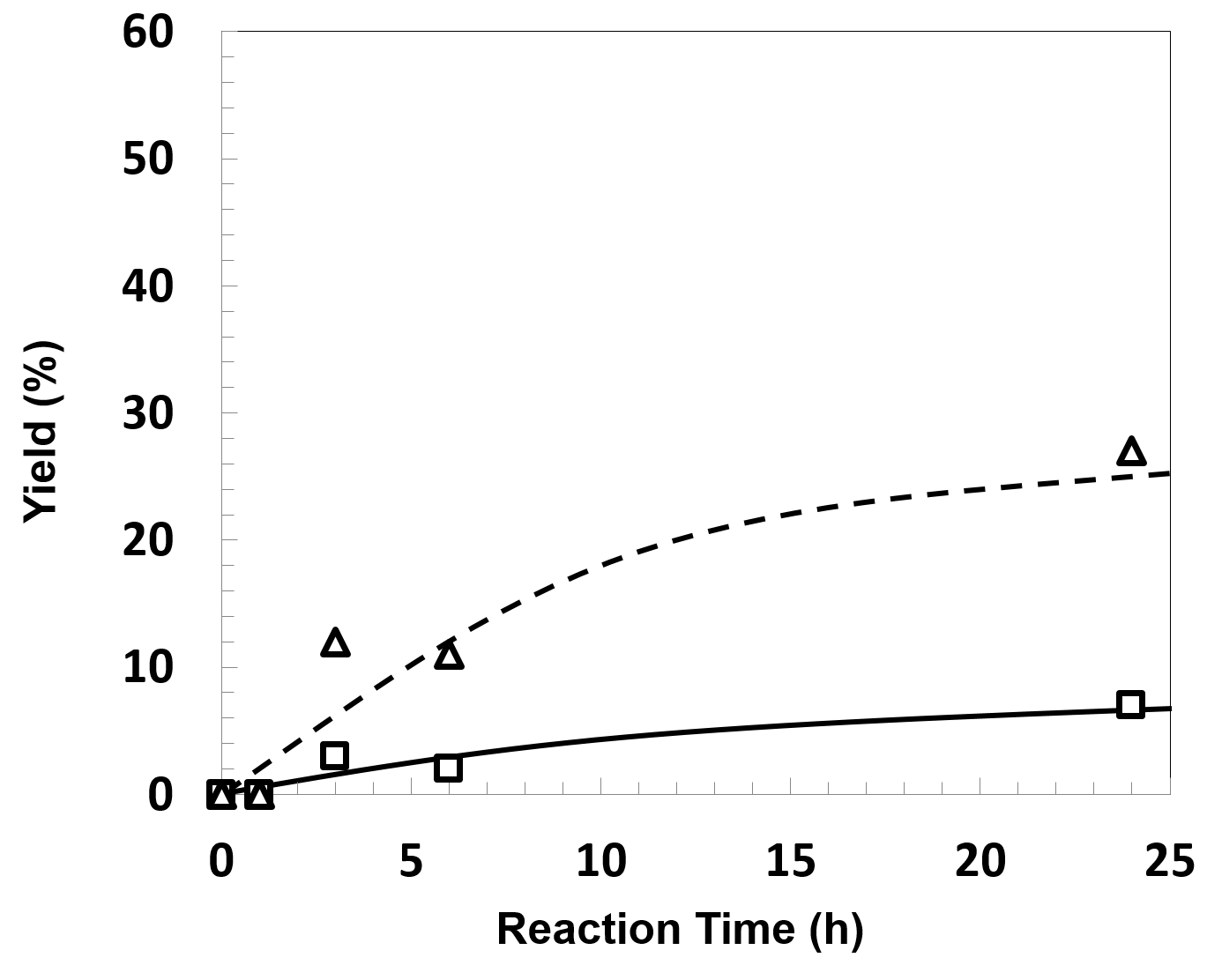


**Figure S2b.** Effect of reaction time on the yields of methyl levulinate (**1**, □) and methyl lactate (**2**, △) using SnBr_4_. Reaction conditions: starch (50 mg), methanol (5.0 mL), SnBr_4_ (0.024 mmol), naphthalene (0.156 mmol), Ar (5 atm), 24 h, 160 ^o^C.

**C, GC-MS Analysis of Oil Components**


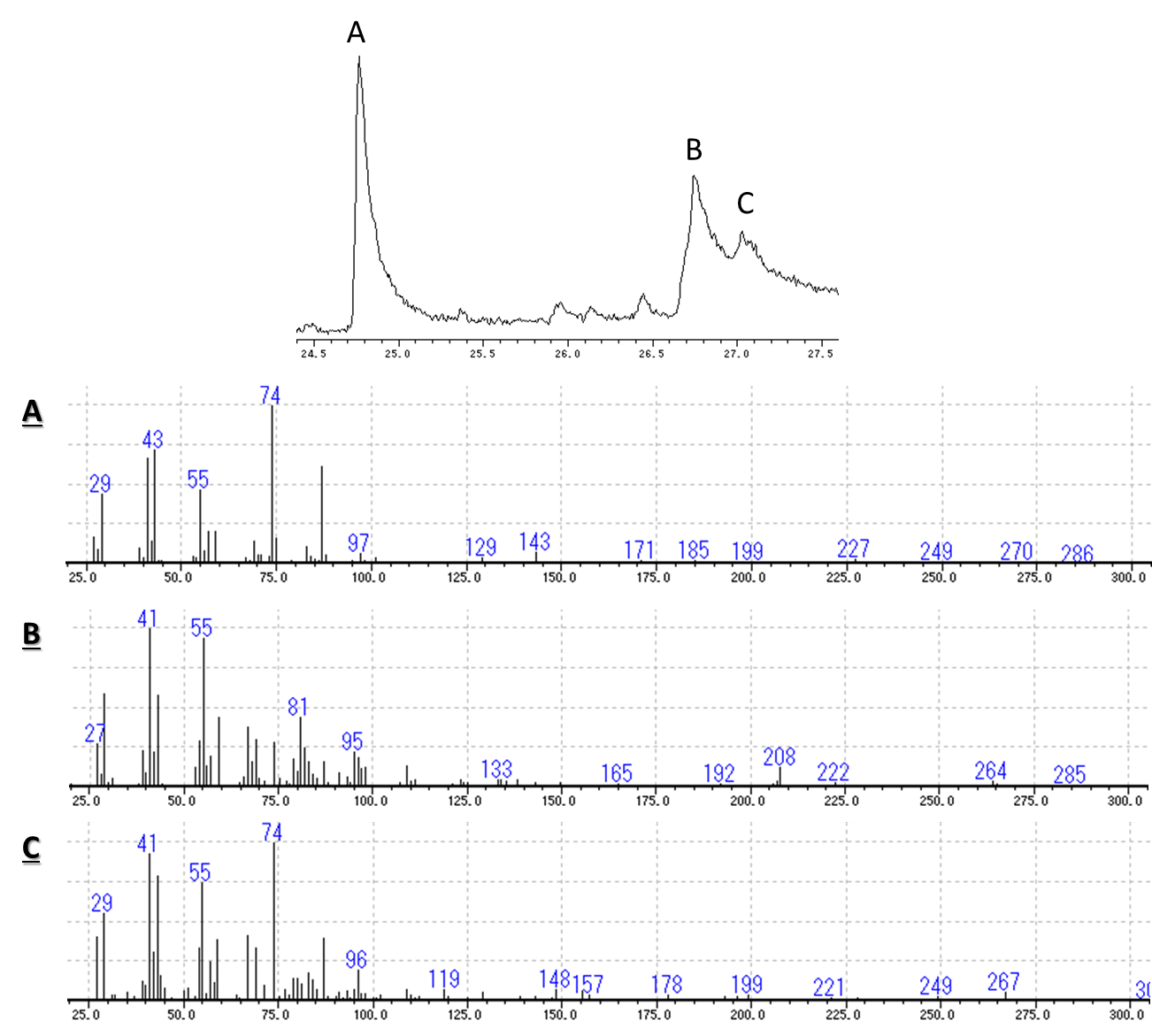


**Figure S3.** Fragmentation patterns of oil components (Table 4, entry 1) as analyzed by GC-MS.

**D, ^1^H NMR Charts of the Reaction Mixture (Table 4, Entries 2 and 3)**


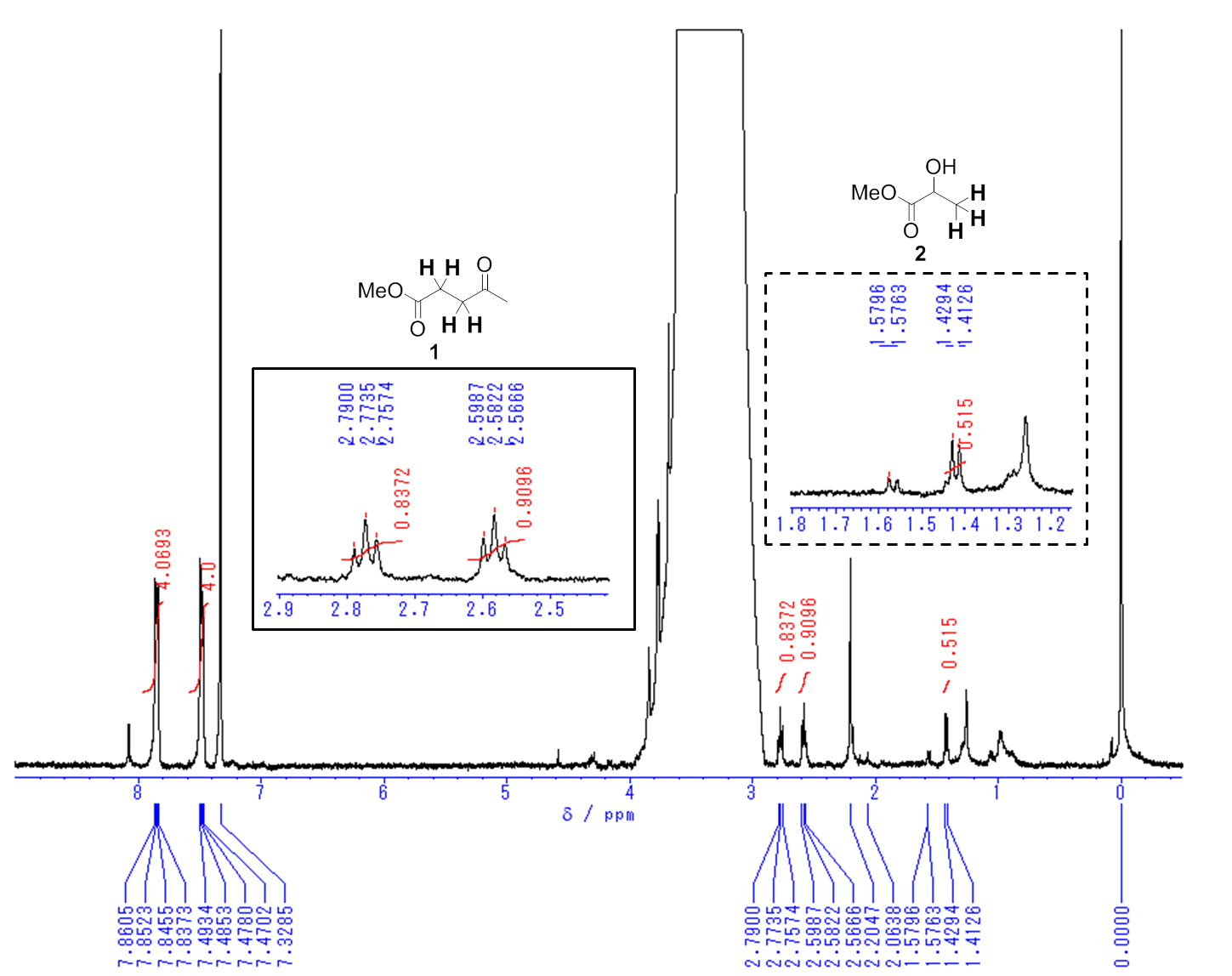


**Figure S4.** A ^1^H NMR chart of Table 4, entry 2.

**
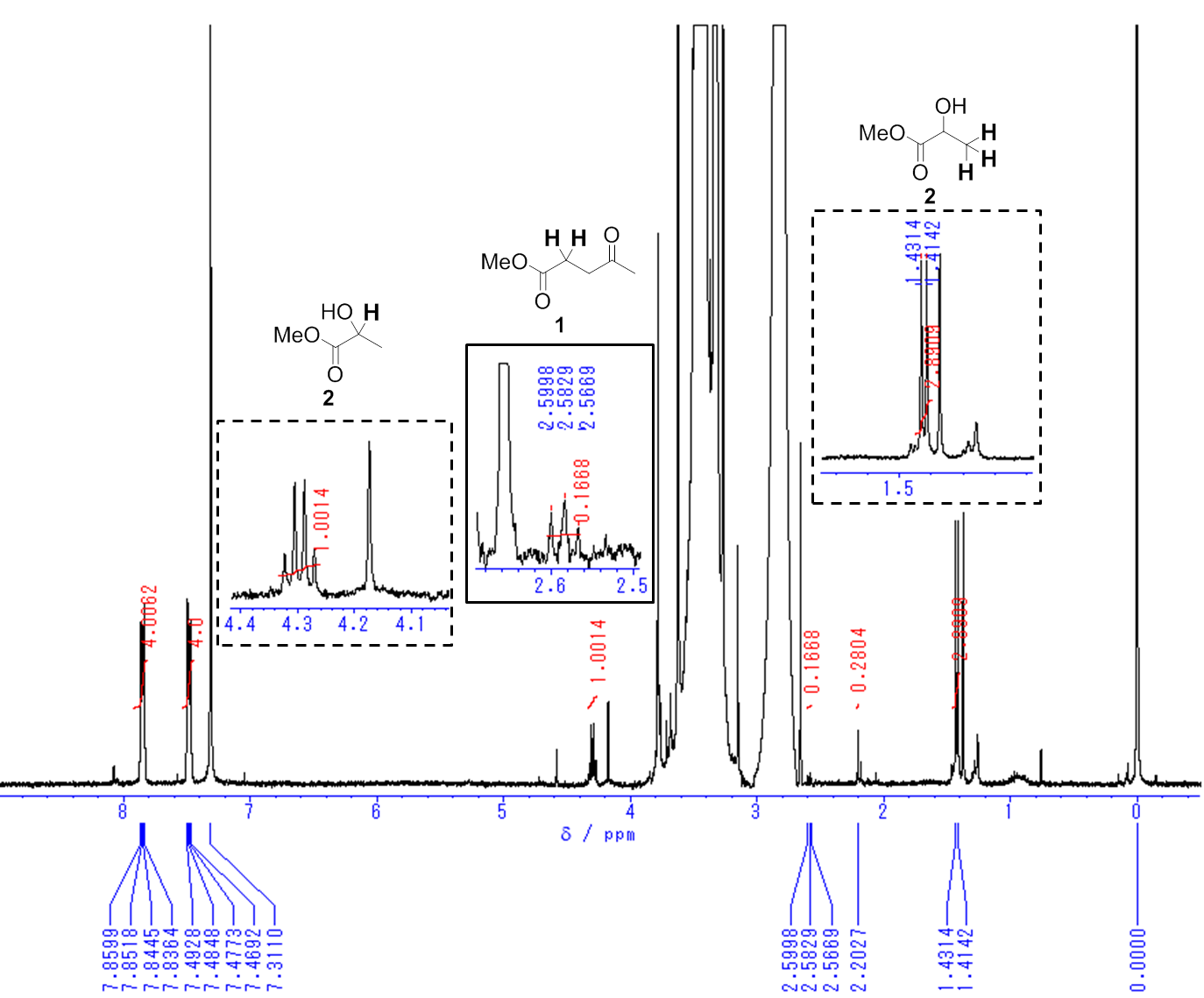
**

**Figure S5** A ^1^H NMR chart of Table 4, entry 3.
